# Differentiating sex and gender in health research to achieve gender equity

**DOI:** 10.2471/BLT.22.289310

**Published:** 2023-08-17

**Authors:** Michelle R Kaufman, Evan L Eschliman, Tahilin Sanchez Karver

**Affiliations:** a Johns Hopkins Bloomberg School of Public Health, Department of Health, Behavior and Society, 624 N. Broadway, Hampton House 257, Baltimore, MD 21205, United States of America.

## Abstract

Effectively tracking progress on initiatives focused on gender equity requires clear differentiation between the terms sex and gender. Sex usually refers to a person’s biological characteristics, whereas gender refers to socially constructed roles and norms. Although both terms are often treated as binaries, gender is a spectrum and sex may include intersex individuals. While the terms are interrelated, they are sometimes conflated or used interchangeably in health data. Their fundamental distinctions, however, have implications for the conduct of research and the design of interventions targeting sex- and gender-based health disparities. We use the example of coronavirus disease 2019 to show how conflating these terms in data collection makes it difficult to ascertain whether disparities in infection rates, morbidity and mortality are determined by sex or gender. Although the exact process of collecting data on sex and gender may need to be adapted for specific contexts, there are steps that can be taken so that health data better reflect the differences between these concepts. Possible actions include using a two-step data collection process to determine both sex and gender of individuals, and encouraging recognition of intersex, third gender, transgender and gender nonbinary people. There also needs to be acceptance and commitment by data collectors and research editors; for example, by using tools such as the Sex and Gender Equity in Research checklist. With clearer distinctions between these foundational terms and how they are used in health data, we can achieve more accurate research findings, better-tailored interventions and better progress towards gender equity.

## Résumé

Pour suivre efficacement les progrès des initiatives centrées sur l'égalité des genres, il est impératif de distinguer clairement les termes «sexe» et «genre». Le sexe fait généralement référence aux caractéristiques biologiques d'une personne, tandis que le genre se rapporte aux normes et rôles socialement construits. Bien que ces termes soient souvent considérés comme binaires, le genre est un spectre et le sexe est susceptible d'inclure les individus intersexués. Tous deux sont étroitement liés; en revanche, ils sont parfois confondus ou employés comme synonymes dans les données relatives à la santé. Pourtant, leurs différences fondamentales ont des conséquences sur la conduite des recherches et l'élaboration d'interventions ciblant les disparités sanitaires fondées sur le sexe et le genre. Dans le présent document, nous citons l'exemple de la maladie à coronavirus 2019 pour montrer que, lorsque ces termes sont assimilés l'un à l'autre dans la collecte de données, il devient difficile d'établir si le sexe ou le genre entraîne des variations au niveau des taux d'infection, de morbidité et de mortalité. Il pourrait s'avérer nécessaire d'adapter la méthode utilisée pour recueillir les données sur le sexe et le genre dans certains contextes spécifiques; néanmoins, il est possible d'entreprendre des démarches pour que les données relatives à la santé reflètent davantage les différences entre ces concepts. Parmi les actions envisagées figure l'usage d'un processus de collecte des données en deux étapes, servant à déterminer tant le sexe que le genre des individus et favorisant la reconnaissance des personnes intersexuées, du troisième genre, transgenres et non binaires. Celles et ceux chargés de récolter les données et de rédiger les recherches doivent également faire preuve d'acceptation et d'engagement, notamment en recourant à des outils tels que la liste de contrôle issue des recommandations sur l'égalité des sexes et des genres dans la recherche (Sex and Gender Equity in Research, SAGER). Mieux comprendre les différences entre ces deux termes essentiels et leur emploi dans les données sanitaires aboutira à des résultats plus précis, des interventions plus pertinentes et davantage de progrès vers l'égalité des genres.

## Resumen

Es necesaria una clara diferenciación entre los términos sexo y género para realizar un seguimiento eficaz del progreso de las iniciativas centradas en la igualdad de género. Por lo general, el término sexo hace referencia a las características biológicas de una persona, mientras que el término género hace referencia a las funciones y normas que dicta la sociedad. Aunque con frecuencia ambos términos se tratan como binarios, género es un espectro y sexo puede albergar personas intersexuales. Aunque estos términos están relacionados entre sí, en ocasiones se confunden o se utilizan indistintamente en los datos sanitarios. Sin embargo, las diferencias fundamentales que existen entre ellos, tienen implicaciones a la hora de llevar a cabo la investigación y el diseño de intervenciones centradas en las disparidades de los datos sanitarios a causa del uso de los términos sexo y género. Utilizamos el ejemplo de la enfermedad de coronavirus de 2019 para mostrar cómo el hecho de confundir estos términos a la hora de recopilar datos, hace que sea más difícil constatar si las disparidades existentes en las tasas de infección, morbilidad y mortalidad están determinadas por sexo o por género. Aunque es posible que sea necesario adaptar el proceso exacto de recopilación de datos sobre sexo y género a contextos específicos, se pueden adoptar medidas para que los datos sanitarios reflejen mejor las diferencias entre estos conceptos. Las posibles medidas incluyen el uso de un proceso de recopilación de datos compuesto de dos pasos para determinar tanto el sexo como el género de las personas, y fomentar el reconocimiento de las personas intersexuales, de tercer género, transgénero y de género no binario. Del mismo modo, es necesario que exista aceptación y compromiso por parte de los recopiladores de datos y de los editores de investigaciones; por ejemplo, mediante el uso de herramientas como la lista de verificación de Sexo e Igualdad de Género en la Investigación. Con distinciones más claras entre estos términos fundamentales, así como en la manera de utilizarlos en los datos sanitarios, podemos lograr resultados de investigación más precisos, intervenciones mejor adaptadas y mejores avances en la igualdad de género.

## ملخص

إن التتبع الفعال للتقدم المحرز في المبادرات التي تركز على المساواة بين النوعين يتطلب تمييزا واضحا بين مصطلحي الجنس والنوع. يشير الجنس عادة إلى الخصائص البيولوجية للشخص، بينما يشير الجنس إلى الأدوار والأعراف المعتادة اجتماعيًا. على الرغم من أن كلا المصطلحين غالبًا ما يتم التعامل معهما على أنهما ثنائيات، إلا أن الجنس عبارة عن فئة، بينما قد يشمل الجنس الأفراد ثنائيي الجنس. على الرغم من أن المصطلحين متربطان، إلا أنه يتم أحيانًا الخلط بينهما أو استخدامها بشكل متبادل في البيانات الصحية. إلا أن الفروق الأساسية بينهما لها آثار على إجراء الأبحاث، وتصميم التدخلات التي تستهدف الفوارق الصحية القائمة على الجنس والنوع. نحن نستخدم مثال مرض فيروس كورونا 2019 لإظهار كيف أن الخلط بين هذه المصطلحات في جمع البيانات يجعل من الصعب التأكد مما إذا كانت الفوارق في معدلات العدوى، والإصابة بالأمراض، والوفيات يتم تحديدها حسب الجنس أو النوع. على الرغم من أن العملية الدقيقة لجمع البيانات حول الجنس والنوع قد تحتاج إلى تكييفها مع أوضاع محددة، إلا أن هناك خطوات يمكن اتخاذها حتى تعكس البيانات الصحية بشكل أفضل الاختلافات بين هذه المفاهيم. تشمل الإجراءات المحتملة استخدام عملية لجمع البيانات مكونة من خطوتين لتحديد جنس الأفراد ونوعهم، وتشجيع الاعتراف بثنائيي الجنس، والجنس الثالث، والمتحولين جنسيًا، والأشخاص غير ثنائيي الجنس. ويجب أيضًا أن يكون هناك قبول والتزام من جانب جامعي البيانات ومحرري الأبحاث؛ على سبيل المثال، باستخدام أدوات مثل قائمة المراجعة الخاصة بالجنس والمساواة بين النوعين في الأبحاث. ومع وجود فروق أكثر وضوحا بين هذه المصطلحات الأساسية، وكيفية استخدامها في البيانات الصحية، يمكننا تحقيق نتائج بحثية أكثر دقة، وتدخلات مصممة بشكل أفضل، وإحراز تقدم أفضل نحو المساواة بين النوعين.

## 摘要

有效跟踪以社会性别平等为重点的举措的进展，需要明确区分生理性别 (sex) 和社会性别 (gender) 这两个术语。生理性别通常是指一个人的生物特征，而社会性别则是指社会所构建的角色和规范。虽然这两个术语通常都被视为二元，但社会性别是一个范畴，而生理性别可能包括双性人。虽然这两个术语是相互关联的，但是在健康数据中它们有时会被合并或互换使用。然而，它们的根本区别会对针对基于生理性别和社会性别的健康差异进行的研究以及干预措施的制定产生影响。我们以新型冠状病毒肺炎为例来说明，在数据收集中混淆这些术语会如何导致难以确定感染率、发病率和死亡率的差异是否由生理性别或社会性别所决定。尽管收集生理性别和社会性别数据的确切过程可能需要根据具体情况进行调整，但可以采取一些措施，以使健康数据更好地反映这些概念之间的差异。可能采取的行动包括使用两步走数据收集流程来确定个人的生理性别或社会性别，并鼓励人们认同双性人、第三性别、跨性别者和非二元性别者。数据收集者和研究人员也需要表示认同并做出承诺；例如，通过使用诸如研究中的生理性别和社会性别平等清单之类的工具。通过更清晰地区分这些基本术语以及它们在健康数据中的使用方式，我们可以获得更准确的研究结果、更有针对性的制定干预措施，并在实现社会性别平等方面取得更好的进展。

## Резюме

Для эффективного отслеживания прогресса в реализации инициатив, направленных на обеспечение гендерного равенства, требуется четкое разграничение понятий «пол» и «гендер». Под полом обычно понимаются биологические характеристики человека, в то время как гендер относится к социально обусловленным ролям и нормам. Хотя оба термина часто трактуются как бинарные, гендер представляет собой спектр, а пол может включать в себя интерсексуальных личностей. Несмотря на то что эти термины взаимосвязаны, в медицинских данных их иногда смешивают или используют как взаимозаменяемые. Однако их фундаментальные различия оказывают влияние на проведение исследований и разработку мероприятий, направленных на устранение гендерных и половых различий в отношении здоровья. Для демонстрации того, как смешение этих терминов при сборе данных затрудняет определение зависимости различий в показателях инфицирования, заболеваемости и смертности от пола или гендера, используется пример коронавирусной болезни 2019 г. Несмотря на то что точный процесс сбора данных о поле и гендере может потребовать адаптации к конкретным условиям, существуют шаги, которые можно предпринять для более четкого отражения в медицинских данных различий между этими понятиями. В качестве возможных мер может использоваться двухэтапный процесс сбора данных для определения пола и гендера человека, а также поощрение признания интерсексов, представителей третьего пола, трансгендеров и гендерно небинарных людей. Также необходимо одобрение и приверженность со стороны сборщиков данных и редакторов исследований, например с помощью таких инструментов, как контрольный перечень «Гендерное равенство в исследованиях». Более четкое разграничение этих основополагающих терминов и их использование в медицинских данных позволит получить более точные результаты исследований, провести более целенаправленные мероприятия и добиться большего прогресса в достижении гендерного равенства.

## Introduction

Several global agencies and world leaders have declared a renewed commitment to addressing gender disparities.^1^^,^^2^ For instance, in 2021 the World Health Organization (WHO) announced multiple commitments to empower women and girls and drive change for gender equality through a focus on reducing gender-based violence, advancing sexual and reproductive health and rights, and supporting health workers.^2^ This announcement builds on efforts since 2009 to integrate gender analysis and actions into the technical work of WHO,^3^ and expands on the commitments to gender equality mentioned throughout WHO’s Thirteenth General Programme of Work 2019–2023.^4^ However, effectively tracking progress on such initiatives requires a clear understanding of two foundational terms: sex and gender. Here we provide a discussion of the distinctions between sex and gender, drawing attention to the risk of conflating these terms and the possible confusion created by using them interchangeably. We further illustrate the utility of distinguishing between sex and gender using data collected on cases of coronavirus disease 2019 (COVID-19). We suggest that clear differentiation of these terms, and systematic data collection and reporting on them, can accelerate efforts towards realizing gender equality and health equity for all.

## Delineating sex and gender

There is a general agreement in the scientific community about sex and gender being two different concepts; sex refers primarily to biological factors, while gender closely aligns to social norms and roles. Definitions of sex often describe it as a construct based on genetics and observed physiological and anatomical sex traits, usually presented as two major categories: male and female.^5^ For example, the United Nations (UN) has used a definition of sex as “the physical and biological characteristics that distinguish males and females.”^6^ However, definitions based on sexual dimorphism do not recognize the rare but diverse set of individuals who are intersex or third sex. These individuals can have a variety of chromosomal compositions beyond the more common XX or XY chromosomes and display a range of sex characteristics, yet are frequently assigned male or female at birth by medical professionals.^7^

Gender, on the other hand, is a social construct that establishes the social norms and roles based on what a society deems appropriate for individuals based on their sex assigned at birth. The UN has used a definition of gender as “[referring] to the roles, behaviours, activities, and attributes that a given society at a given time considers appropriate for men and women… [that] are socially constructed and are learned through socialization processes.”^6^ However, there can be many ways in which an individual may not conform to the prevailing gender norms, such as when an individual feels an inherent sense of gender identity that differs from their sex assigned at birth. Gender identity can be defined as “someone’s personal and deeply felt internal sense of the self, which may or may not correspond with the person’s physiology or designated sex at birth.”^6^^,^^8^

## Acknowledging diversity

Capturing data on sex and gender is necessary for measuring and analysing the variations and complexities related to these factors, and their influences on health within and across different settings. We also need clarity around how and to what extent the data collected differentiate between the concepts of sex and gender. The biological and social realities that accompany one’s sex and gender affect an individual’s health and well-being in many interrelated and complex ways.^9^ For example, sex-related biological differences can result in different manifestations of disease, including variations in symptoms or incidence – such as the much higher incidence of breast cancer among females. Gender, on the other hand, is a social determinant of health in how it shapes social norms dictating roles, responsibilities and access to power and opportunities that ultimately influence the health and well-being of individuals. In most societies and cultures, gender involves “differences and inequalities between women and men in responsibilities assigned, activities undertaken, access to and control over resources, as well as decision-making opportunities.”^6^ Such differences result in relationships, communities and institutions and policies also being gendered. While the incidence of breast cancer among females is a sex-related issue, having the agency and resources to access care for breast cancer is a gender-related issue.

Challenges in differentiating between sex and gender in the collection of health data also arise from how these constructs often – but not necessarily – overlap at the individual level, for example, in the experience of transgender or nonbinary individuals. Furthermore, context-specific considerations can shape the recognition and discussion of sex and gender diversity. Because sex and gender are related yet distinct concepts,^10^^,^^11^ data that are intended to determine an individual’s gender can more accurately be capturing their sex – or vice versa. For example, assignment of a sex does not mean that a person experiences gender in the same ways as everyone else assigned that sex; a person can be assigned female sex at birth but can hold a non-female gender identity. This example is one way that data reflecting sex assigned at birth are limited in their ability to reflect individuals’ experience of gender. Conversely, if data intended for capturing gender and the associated social forces are denoted as capturing sex, this can confuse data interpretation, particularly when disparities are highlighted. In such cases, disparities noted by sex (which include hormone levels and differences in body structure) may be mistaken for disparities that are caused by gendered factors (such as environmental exposures, care-seeking behaviours or health risk behaviours).

Efforts towards a clear differentiation between sex and gender in data collection may also have to consider cultural variations or laws on gender diversity that may necessitate a particular approach to research and data collection in a given setting. For instance, if identifying as transgender is illegal, or if being known to be transgender could result in harm to an individual, data that differentiate between sex and gender may be impossible to collect. Whenever possible, efforts towards an understanding of how sex and gender (and gender identity) are distinct or related is important when justifying the use of different approaches to collecting and analysing data.

Without clear differentiation between how sex and gender influence health outcomes, the pathway for addressing disparities in health outcomes remains unclear. Precision in the use of each construct could focus attention on how best to direct health policy and programming. For example, data on sex at birth is often more accurately construed as an individual variable that requires individual-level intervention. Gender, on the other hand, could be related to inequitable gender norms, social roles or gender discriminatory policies that may require longer term, multilevel interventions or changes in social norms.

In addition, a greater understanding of the way sex characteristics and gender identities beyond the binary (female or male; woman or man) impact the health and well-being of individuals is necessary to address the root causes of health disparities and to meet the unique and diverse needs of individuals. For example, although most countries’ laws and policies provide legal recognition for only binary sex and gender categories, such as on birth and death registrations or identity documents, other countries (including India and Thailand^12^^,^^13^) allow for other or intersex or third gender categories and are more successful at collecting more accurate data on all citizens. Although these additional categories can still group together diverse sets of individuals, the categories can be a starting point in allowing a country to identify and address disparities in health outcomes for all its citizens.

## Example: COVID-19

The importance of distinguishing between sex and gender in the collection, analysis and use of health data is illustrated by the data generated during the COVID-19 pandemic. For example, biologically, males have been reported to have more severe symptoms and higher mortality from COVID-19 than females.^14^ Females, however, have been shown to be more likely to develop post-COVID-19 condition (long COVID) compared with males.^15^^,^^16^ On the other hand, an analysis of how sex- or gender-related factors influence COVID-19 outcomes such as infection, symptom severity and mortality would raise questions around how certain gendered occupations, such as health-care work, may contribute to greater exposure to the virus. Gender norms which place women more often in caretaking roles such as health workers, teachers and caregivers for children and older adults, may have contributed to greater increases in anxiety and mental distress for women than men during the early stages of the pandemic.^17^ Furthermore, research using data that included categories for nonbinary and transgender individuals has documented how discrimination in health services prevented such individuals from seeking early testing and treatment for COVID-19 (Kaufman MR et al., Johns Hopkins Bloomberg School of Public Health, unpublished data, 2023).

Even so, COVID-19 data on sex and gender are limited. Our scoping review of the social determinants of COVID-19 symptom presentation and outcomes found that studies often conflated the terms sex and gender (Kaufman MR et al., Johns Hopkins Bloomberg School of Public Health, unpublished data, 2023). As is the case in health data collection and analysis more broadly, mislabelling the concepts or using them interchangeably creates challenges in differentiating between sex and gender and in interpreting the data. In this example of COVID-19, lack of clarity about the construct being employed in an analysis limits our understanding of a study’s findings. We would not know if the findings were related to sex; for example, if there is a true biological determinant to increased COVID-19 mortality in males. Or the findings could be related to gender; for example, if gender inequality and its associated gender roles and norms puts women at increased risk of exposure to the virus. The findings might even be related to both sex and gender; for example, we would not know if females were more physiologically susceptible to long COVID, or if rates of diagnosis were higher among women because of gendered expectations of seeking health care more frequently.

## Moving forward

We are continuing to expand our understanding of how health outcomes are influenced by sex and gender, and the ways in which these constructs intersect. Future strategies should therefore ensure that the distinctions between these two constructs are recognized and implemented in health research. This process would take concerted effort by all individuals and institutions that collect, analyse and publish health data. To make progress, we first need to place an emphasis on training health researchers and other professionals on the differences between sex and gender, and how to adequately capture and display these differences in the collection, analysis and use of health data. We call on researchers to think critically about the influence of sex and gender on health behaviours and outcomes – and other factors that may contribute to health, such as education, employment and public policy. This process includes precision in articulating research questions, and understanding when it is appropriate to use a sex- versus gender-based measure or apply a sex- versus gender-based analysis.^18^^,^^19^

The current debate aligns with reports issued by various medical and health research institutions, such as the guidelines for reporting of Sex and Gender Equity in Research (SAGER) published by the European Association of Science Editors;^20^ the Psychological Society of South Africa sexual and gender diversity position statement;^21^ and a 2022 report from the United States National Academies of Sciences, Engineering and Medicine.^5^ Each of these organizations urges data collection, analysis and reporting that relies on distinct sex and gender measures, and better reporting of the way categories are defined and measured and data are collected. Moreover, all these reports caution about conflating sex and gender and encourage acceptance and recognition of transgender, nonbinary and gender-diverse individuals.

This call for transparent and clear definitions of how data by sex and gender are collected and analysed also extends to journal editors and associated reviewers.^20^ These individuals have the power to ask for more clarification on a measure, and can provide guidance on the correct use of terms. In some cases, this guidance may include offering editing for more accurate use of terms and asking for reporting of data by sex or gender. Tools such as the SAGER checklist have been developed to aid editors.^19^ We need to address the challenges and recognize the social and health inequities often experienced by people based on their sex or gender. To succeed, we need a shared commitment from all people involved in capturing, analysing and publishing related health data.

One move towards ensuring the most meaningful measurement of sex and gender could be to use a two-step process in health data collection.^5^ This process requires capturing data on sex assigned at birth, and separately capturing data on self-reported gender. For example, a cisgender woman could report being assigned female at birth in the first step, then report her gender as woman in the second step; whereas a transgender woman could report being assigned male at birth in the first step, then report her gender as woman in the second step. This two-step process could be a standard approach for researchers to acknowledge the differences in these constructs – similar to the collection of key sociodemographic characteristics such as age, education and marital status – and allow for adaptations to specific contexts. Furthermore, this two-step process would not only allow for better characterization of sex and gender as different constructs, but also provide an opportunity to explore the intersectionality of sex and gender when necessary.

This approach can yield great benefits in providing more accurate and valid data on people whose gender identity is different from their sex assigned at birth. However, in some contexts, these groups may be stigmatized or even subject to criminal prosecution. The use of this two-step process should therefore also consider issues of safety and confidentiality. Furthermore, these groups are themselves diverse, and greater understanding of the biological versus social impacts on their health and well-being may require additional data points than only sex and gender, such as data on hormone use or gender expression.

Nevertheless, using such guiding principles will help efforts to document how data collection has been done relative to these suggested best practices. This approach will allow further investigation and understanding of the important roles that sex and gender play in the lives of individuals, and how these roles affect health and well-being at the individual and population levels. This distinction between sex and gender would also offer greater insights for policy-makers and other relevant people and organizations about how to invest in programmes and policies that address inequalities in global health due to sex and gender.

## Conclusion

As data collection efforts continue to highlight and reveal gender disparities and health inequities worldwide, consistency in the use of the terms sex and gender, and differentiating between the two, is necessary. At the same time, given some of the pragmatic and ethical concerns of sex and gender data collection across different contexts, complete congruence in how these data are collected is likely impossible. In the meantime, while working towards consistency as much as possible, data collectors, researchers and those disseminating data findings can commit to clearly documenting the definitions, procedures and reporting related to sex and gender. With careful attention to the distinctions between these terms and how they are used in health data, research findings will become more accurate and health interventions better tailored towards improving gender equity.
